# Detection of the Microbial Composition of Some Commercial Fermented Liquid Products via Metagenomic Analysis

**DOI:** 10.3390/foods12193538

**Published:** 2023-09-22

**Authors:** Cansu Çelik Doğan, Hafize Tuğba Yüksel Dolgun, Serkan İkiz, Şükrü Kırkan, Uğur Parın

**Affiliations:** 1 Food Technology Program, Food Processing Department, Vocational School of Veterinary Medicine, Istanbul University-Cerrahpaşa, 34320 Istanbul, Türkiye; 2 Department of Microbiology, Faculty of Veterinary Medicine, Aydın Adnan Menderes University, 09010 Aydın, Türkiye; tugba.yuksel@adu.edu.tr (H.T.Y.D.); skirkan@adu.edu.tr (Ş.K.); uparin@adu.edu.tr (U.P.); 3 Department of Microbiology, Faculty of Veterinary Medicine, Istanbul University-Cerrahpaşa, 34320 Istanbul, Türkiye; ser@iuc.edu.tr

**Keywords:** metagenome, microbiome, hardaliye, kombucha, shalgam juice (a fermented turnip juice drink), pickle juice, grape vinegar, 16S rRNA, plant growth promotion, probiotics

## Abstract

The fermented liquid sector is developing all over the world due to its contribution to health. Our study has contributed to the debate about whether industrially manufactured fermented liquids live up to their claims by analyzing pathogens and beneficial bacteria using a 16S rRNA sequencing technique called metagenomic analysis. *Paenibacillus*, *Lentibacillus*, *Bacillus*, *Enterococcus*, *Levilactobacillus*, and *Oenococcus* were the most abundant bacterial genera observed as potential probiotics. *Pseudomonas stutzeri*, *Acinetobacter*, and *Collimonas*, which have plant-growth-promoting traits, were also detected. The fact that we encounter biocontroller bacteria that promote plant growth demonstrates that these organisms are widely used in foods and emphasizes the necessity of evaluating them in terms of public health. Their potential applications in agriculture may pose a danger to food hygiene and human health in the long term, so our data suggest that this should be evaluated.

## 1. Introduction

The fermented liquid sector is growing globally due to increased demand from consumers. For quite some time, they have been considered to have health-promoting attributes [1]. There are many fermented liquids with low alcohol or non-alcohol content that are consumed by people all around the world. Most countries have their own traditional fermented liquids and their microbiological structures have been analyzed due to their potential health-promoting properties and probiotic characteristics [2,3]. Pickle juice, grape vinegar, shalgam juice (a fermented turnip juice drink), kombucha, and hardaliye are the liquid groups that have been studied in this research.

Fermented foods have become a remarkable issue with their nutritional and probiotic contents. The development of effective probiotic products depends on the studies to be carried out in this field. Pickle juice is rich in probiotics, vitamins, and minerals. The 16S rRNA sequencing technique detected that the microbial composition of pickle juice comprises the *Lactobacillus*, *Pediococcus*, and *Enterococcus* strains. Vinegar production is a two-stage process including alcohol and acetic acid fermentation. *Lactococcus, Oenococcus,* and *Acetobacter* were detected in apple cider vinegar as the dominant bacteria. Shalgam juice is a traditional Turkish fermented beverage made of black carrot, rock salt, sourdough, bulgur wheat flour, turnip, and water. Since shalgam juice is produced through lactic acid fermentation, lactic acid bacteria (LAB) play an important role in its formation. *Lactiplantibacillus plantarum* and *Lentilactobacillus buchneri* were found to be dominant in shalgam juice. Hardaliye is a locally produced fermented beverage made of red grapes or grape juice with the addition of crushed mustard seeds and benzoic acid. In industrial production, benzoic acid can be used as a preservative in mustard. *L. paracasei* subsp., *paracasei*, and *L. casei* subsp. *pseudoplantarum* were found to be dominant in hardaliye. Kombucha is a sweetened black tea beverage that is fermented from the kombucha mushroom. The fungal and bacterial diversity of kombucha has been shown by many culture and sequencing methods. *Acidobacteria*, *Actinobacteria*, *Armatimonadetes*, *Bacteroidetes*, *Deinococcus-Thermus*, *Firmicutes*, *Proteobacteria*, *Verrucomicrobia*, and *Gluconacetobacter* create the microbial diversity of kombucha as a major bacterium in multiple results. Respective bacteria have a key role as probiotics in dietary adjustments, have many significant health benefits for the gut microflora, and also contribute to the production processes of fermented liquids [2,3,4,5,6,7,8,9,10].

Conventional methods such as culture-dependent isolation may restrict the potential microbiological load; therefore, a large number of microorganisms could not be isolated [11]. Also, polymerase chain reaction (PCR) and quantitative PCR (qPCR) were reported to cause a limited number of validated primers for targeted genes, to have a low throughput, and pose amplification bias [12]. To fully understand the distribution of the bacterial community, researchers prefer novel techniques such as metagenomic analysis encompassing 16S rDNA sequencing and shotgun sequencing [13,14].

The main objective of this research was to reveal the microbial composition and the distribution of probiotics among some packaged fermented liquid product groups; it was also aimed to determine the highest number of bacterial communities that dominate the microbiome of kombucha, hardaliye, shalgam juice, grape vinegar, and pickle juice samples by the 16S rRNA sequencing technique called metagenomic analysis. While investigating the microbial diversity of our products, which have different production processes; we aimed to reveal the probiotic content of this bacterial population, so this work will contribute to a deeper understanding of whether commercial fermented liquids contain the probiotics they claim and how reliable the ready-to-eat products are.

## 2. Materials and Methods

### 2.1. Sampling

Four different brands of hardaliye and five different brands of kombucha, shalgam juice, grape vinegar, and pickle juice were directly collected from retail and online stores in several cities in Türkiye between January and March 2021. According to the information on the label, some of them were produced in a traditional way and some of them were manufactured using industrial process techniques. They were transmitted to Aydın Adnan Menderes University, Faculty of Veterinary Medicine, Department of Microbiology Molecular Laboratories, under the cold chain. For the purpose of enhancing the reliability and validity of the research, four subsamples were taken from different bottles of each brand; thus, a total of 96 samples were collected to be analyzed (Table 1). The data, including the production date, the expiration date, and the date the samples were purchased, were saved.

### 2.2. DNA Extraction

The DNA extractions of 96 samples were carried out using the MagAttract HMW DNA Kit (Qiagen^®^, Hilden, Germany). A Qubit 2.0 fluorometer (ThermoFisher Scientific^®^, Waltham, MA, USA) was used to determine the densities of the DNA samples. Five food groups were evaluated in different pools. A total of 24 DNA pools were created by mixing the DNA extracts of the same brand at equal concentrations. The DNA pools were stored in a deep freezer at −20 °C for use in microbiome analysis.

### 2.3. Metagenomic Sequencing

A Qubit 2.0 fluorometer (ThermoFisher Scientific^®^) was used to determine the densities of the pooled DNA samples. Then, 16S PCR was performed using tailed 27F and 1492R universal primers. The 16S PCR reactions in the 50 μL volume contained Primer 27F (2 µL), Primer 1492R (2 µL), Template DNA (21 µL), and LongAmp Taq 2X Master Mix (25 µL) (New England Biolabs Inc., Ipswich, MA, USA). The PCR amplification conditions were as follows: initial denaturation at 95 °C for 1 min followed by 30 cycles of denaturing at 95 °C for 20 s and later annealing at 55 °C for 30 s; then, the extension step at 65 °C took place for up to 1.15 min followed by a single extension step at 65 °C for 5 min and the last step ended at 4 °C. AMPure XP (Beckman Coulter^®^, Brea, CA, USA) was used to purify the products after PCR. After the purification, the barcoding PCR process was performed using the PCR Barcoding Expansion Kit 1-96 (EXP-PBC096) (Nanopore^®^, Oxford, UK). The products obtained after the barcoding process were again purified with AMPure XP. After the DNA densities were measured, all products were collected in an Eppendorf tube to create the final barcoded DNA library. The DNA repair PCR was performed in accordance with the protocol of the barcoding kit in order to eliminate damage to the DNA. The product obtained after the DNA repair PCR was purified again with AMPure XP and the adapter-binding PCR process started. The Ligation Sequencing Kit (SQK-LSK 109) (Nanopore^®^) for adapter ligation PCR was performed as specified in the protocol and incubated at room temperature for 10 min. Then, the product was purified with AMPure XP and the loading mix specified in the protocol was created to load the product into the instrument. After the Flowcell (Flow Cell R9 Version Nanopore^®^) was checked with the MinKNOW program (Nanopore^®^), the Flow Cell Priming Kit (Nanopore^®^) was used for the loading process and the loading process was completed as specified in the protocol. The Flow Cell Priming Kit (Nanopore^®^) was used for the loading process and the loading process was carried out according to the protocol. After the loading process, a 24-h sequencing protocol was performed in the MinKNOW program which was placed on the Flow Cell MinION (Nanopore^®^) device.

### 2.4. Statistical Analysis

After sequencing, the results obtained in the fast5 format were converted to the fastq format (basecalling and multiplexing) using Guppy v3.1.5 software. The barcode and adapter sequences were cleaned using Porechop v0.2.3 software and reads of 1350–1550 bp length were filtered. Cleaned reads were analyzed with a customized workflow using the Mothur v.1.39.5 platform. Sequences were deconstructed and aligned and OTUs were formed by clustering reads with greater than 99% similarity by measuring the distances between them with the similarity matrix. The taxonomic annotations were performed by comparing OTUs according to the RDP 16S rRNA database and statistical results were obtained by associating the OTUs detected as the same genus. Graphs were created with various statistical analyses using the Minitab and R programs according to the organisms whose OTUs matched, their quantitative values, and the metadata of the samples. All the reads were filtered and cleaned and the OTUs were then created. The taxonomic annotations were performed by comparing the created OTUs according to the RDP 16S rRNA database and statistical results were obtained by associating the OTUs detected as belonging to the same genus. Data management and analysis were performed using the Minitab and R programs.

## 3. Results and Discussion

Our study focused on industrial products manufactured using different techniques and sold both in retail and online. There were many studies related to the metagenomic analysis of homemade foods but studies with packaged products were insufficient. Thus, our choice of packaged products such as kombucha, hardaliye, shalgam juice, grape vinegar, and pickled juice appears to be reasonable in terms of ensuring a new insight about the distribution of the bacterial community in packaged liquids. The microbiota composition of five different fermented liquids was analyzed using next-generation sequencing (NGS)-based 16S rRNA microbiome analysis techniques.

The microbial distributions were determined at the phylum, family, genus, and species levels. The 16S rRNA gene-based results showed that there were nine different bacterial phyla; however, two of them were found to be the most abundant: Firmicutes and Proteobacteria. Pickle juice had the highest level of Firmicutes, followed by shalgam juice, and grape vinegar. Conversely, Proteobacteria were found in the highest concentrations in kombucha, followed by hardaliye, and grape vinegar. In this study, we mostly presented the sequences at the genus level because NGS is reliable up to the genus level since most false positives can be eliminated at the genus level but not at the species level. Hence, NGS is the most powerful option for studying bacterial diversity in environmental samples [15].

**Figure 1:**
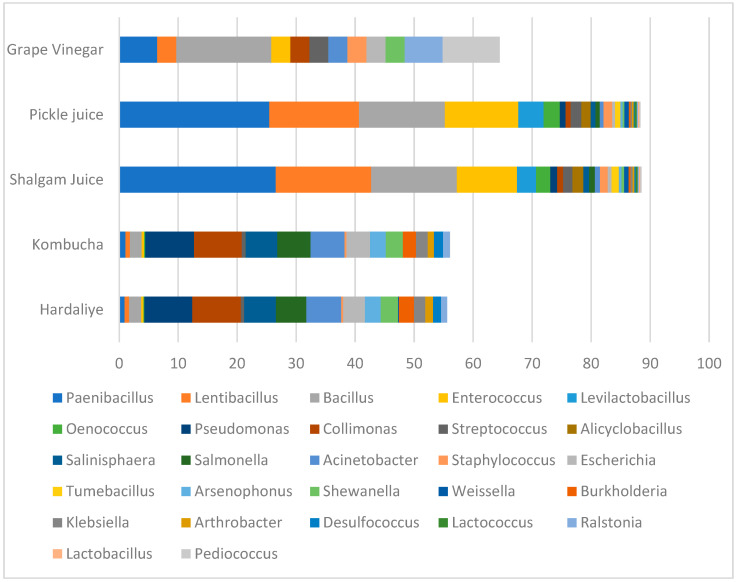
Comparing the relative abundance of the main OTUs across samples.

In grape vinegar samples, *Bacillus*, *Pediococcus*, *Paenibacillus*, *Ralstonia*, *Lentilactobacillus*, *Lentibacillus*, *Enterococcus*, *Streptococcus*, *Acinetobacter*, and *Collimonas;* in shalgam juice and pickle juice samples, *Paenibacillus*, *Lentibacillus*, *Bacillus*, *Enterococcus*, *Levilactobacillus*, and *Oenococcus*; in kombucha and hardaliye samples, *Collimonas*, *Pseudomonas*, *Acinetobacter*, *Salinisphaera*, and *Salmonella*, appeared, respectively, and dominantly (Figure 1). Our study also revealed a number of sub-dominant phyla and genera not previously associated with kombucha, hardaliye, shalgam juice, grape vinegar, or pickle juice. This is an interesting finding as it is contrary to what we know from previous studies that carried out the fermentation step in their laboratory. While *Zygosaccharomyces*, *Gluconacetobacter*, *Komagataeibacter*, *Acetobacter*, *Lactococcus*, and *Lactobacillus* were detected as the dominant genera in some studies [1,5,8,10,16,17,18], they were detected at very low levels or were entirely missing in our samples.

**Figure 2:**
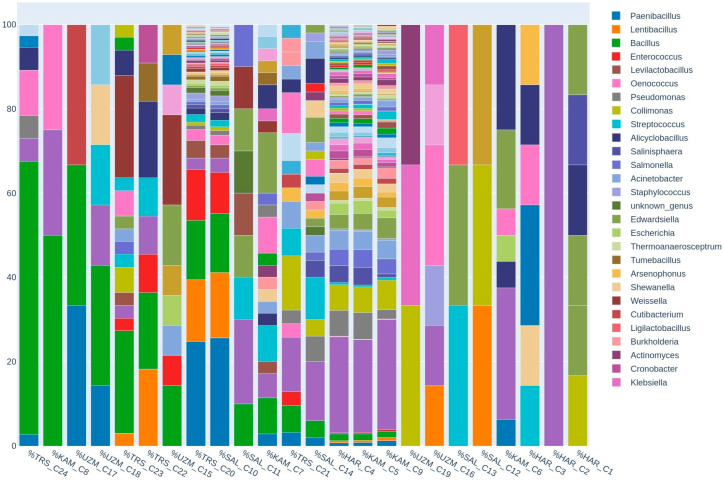
The order of samples from most to least containing the genus Bacillus. UZM: Grape Vinegar, HAR: Hardaliye, KAM: Kombucha, TRS: Pickle Juice, SAL: Shalgam Juice.

**Figure 3:**
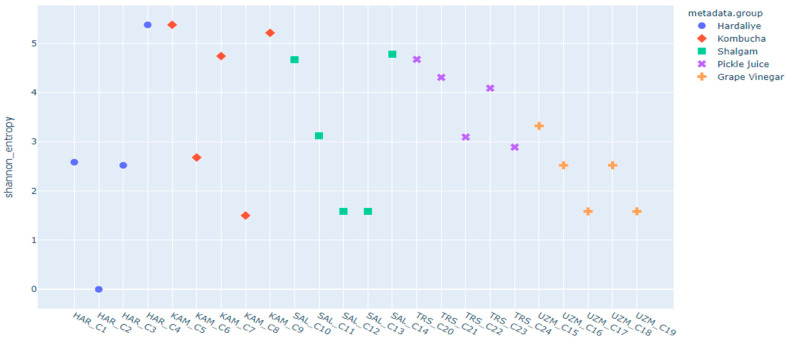
Shannon entropy diversity. UZM: Grape Vinegar, HAR: Hardaliye, KAM: Kombucha, TRS: Pickle Juice, SAL: Shalgam Juice.

Commercially produced fermented liquids with labels that claim that the product contains probiotics are likely to have variable microbial compositions. Zhang et al. [19] stated that microbial resources in traditional fermented foods all around the world showed polymorphisms associated with their geographical distribution. The results may also vary between homemade and industrially produced fermented liquids [20,21]. Other factors that may vary the presence of microbiota in commercial fermented liquids should be considered. The quantity of microorganisms present at the time of consumption, even in the absence of the heat or separation phase, can be affected by the nutrient composition, the storage environment, and the age of the food [22]. For instance, although neither pasteurization nor food additives were used in the samples of C6, C8, C16, or C17, the microbial diversity was not as high as expected, as shown in Figure 2. Shannon entropy diversity can be seen in Figure 3, which shows the similarities and alpha diversity of groups among themselves and each other. According to Shannon entropy diversity, the samples of C4, C5, C9, C10, and C20 were detected as having the highest microbial diversity.

Some *Bacillus* species, mostly used as probiotics in food and beverage production, were encountered at the highest rate in shalgam juice, which grows underground, in pickle juice, which is made from root vegetables, and also in grape vinegar in our study. Although most of the grape vinegar samples (C15, C17, and C18) did not have the highest microbial diversity, they were detected as having potential probiotics at a high rate, as shown in Figure 2, which demonstrates the order of samples containing the genus *Bacillus*.

**Figure 4:**
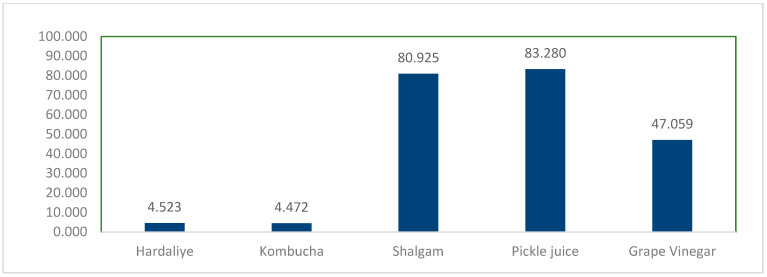
The distribution of the Bacilli class in product groups.

Pittia and Antonello [23] stated that the sugar content of fermented products has the potential to reduce microbial viability by decreasing water activity. According to the labels, grape vinegar, hardaliye, and kombucha contain the highest amount of sugar, whereas pickle juice and shalgam juice have less. As a consequence, the following percentages are the rate of class Bacilli in pickle juice (83.28%), shalgam juice (80.92%), grape vinegar (47.05%), hardaliye (4.5%), and kombucha (4.47%) (Figure 4).

In the study by Kunene et al. [24], it was stated that the genera *Pediococcus* and *Lactobacillus* were identified at low frequencies in plant-based foods. Except for the fact that we found a higher concentration of them in grape vinegar (8.8%), their result was confirmed in our study by finding very low levels of *Pediococcus* in other types of liquid products. Ryu et al. [25] found that *Enterococcus* and *Acinetobacter* were present in high relative abundance in their traditionally fermented foods, similar to our results. However, the results showed variance in our study between food groups. For instance, *Enterococcus* was found at the highest level in shalgam juice (9.8%), pickle juice (12%), and grape vinegar (3%) but not in hardaliye or kombucha, while *Acinetobacter* was detected at the highest level in hardaliye (4.4%), kombucha (4.3%), and grape vinegar (3%) and not in shalgam juice or pickle juice samples. *Bacillus velezensis* is a novel species that can be used in the fermentation industry for its antifungal effect [26] and 2.9% of the microbial composition was detected as *B. velezensiz* in shalgam juice and pickle juice in our research.

Free-living microorganisms that promote plant growth and are used as biological fertilizers are called plant growth-promoting rhizobacteria (PGPR) [27]. Related studies show that there has been a gradual increase in the number of bacteria that have been reported to enhance plant growth. In the agriculture and food industries, there has been an interest in replacing chemical fertilizers, pesticides, and supplements with plant growth-promoting bacteria [28]. Plant growth-promoting bacteria originating from water and soil were also detected in our research, as summarized in Table 2. With respect to our study, *Collimonas* spp. (6.1%) and *Pseudomonas stutzeri* (5%) took the highest share in hardaliye and kombucha, respectively. This is mainly due to the environment where the raw materials grow, as described by González-Arenzana et al. [29]. *Arthrobacter* is an inhabitant of soil, air, the surface of plants, ready-to-use vegetables, and wastewater and was found in hardaliye samples (Table 2). *Arthrobacters* are found in ready-to-use vegetables [30]. As phytopathogens, *Ralstonia* spp. were detected in grape vinegar samples (5.8%). The food industry uses halophilic bacteria to benefit from their enzyme production. Such enzymes from halophiles can also be used in fermented foods. In addition, 4% of the reads obtained from hardaliye and kombucha belonged to *Salinisphaera*-related OTUs. *Salinisphaera* sp. strain LB1 is gaining attention as a halo-acidophilic bacteria since it can produce enzymes that are necessary for industrial fermentation processes. The enzymatic activity of *Salinisphaera* sp. strain LB1 can be effective in conditions of low pH and high concentrations of salt and their strains were isolated from surface water [31]. *Lentibacillus* spp. were reported to grow during fermentation. Similar to the isolation of *Lentibacillus* spp. from fermented liquids reported in previous studies [32,33,34,35], they were also seen at the highest levels in our samples of shalgam juice (15.6%), pickle juice (14.8%), and grape vinegar (3%). The only species belonging to this genus was detected as *Lentibacillus* sp. CBA3610 in our samples. Also, *Paenibacillus barcinonensis* and *Paenibacillus mucilaginosus*, used in agricultural and industrial production processes, were encountered in shalgam juice, pickle juice, and grape vinegar samples [36,37].

The processes of commercial liquid products require various technological steps. Amongst them, the thermal process, fermentation, addition of substances with antimicrobial properties, storage conditions, and shipping temperature are the ones that have the most ability to damage the probiotics [49]. For instance, after blending, pumping, the addition of ingredients, and pasteurization processes, the loss of viability of probiotics, including *Lactobacilli* and *Bifidobacterium*, was indicated by many investigators [50,51,52].

Another issue regarding commercial fermented foods and beverages is that they may not contain declared probiotic bacteria and may not be in agreement with the label statements [53]. According to the labels, some of our products are sold as pasteurized and/or have preservatives added, while others have neither been heated nor have chemicals applied. In our study, pasteurized products (C2, C3, C12, C13, and C19) were detected as having the least microbial diversity, regardless of whether food preservatives had been added. Conversely, unpasteurized products (C4, C5, C9, C10, and C20) were found to have the highest bacterial diversity (Figure 2 and Figure 3). This finding is also supported by the study of İyiçınar [54], which was conducted with shalgam juice, and which discovered that pasteurization reduced the number of LAB. Ding and Shah [52] stated the same inhibitory effect of pasteurization.

**Figure 5:**
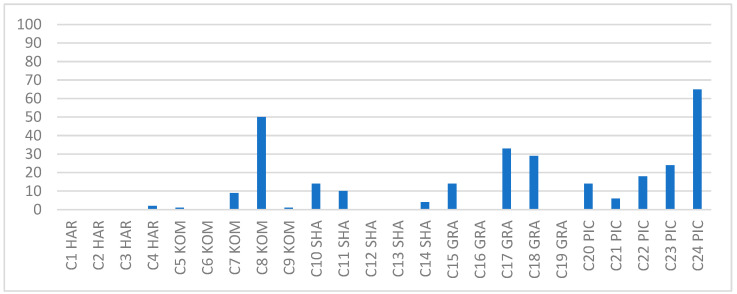
The percentage of the Bacillus genus in each sample. GRA: Grape Vinegar, HAR: Hardaliye, KOM: Kombucha, PIC: Pickle Juice, SHA: Shalgam Juice.

Microbes can be highly sensitive to food additives. Preservative agents reflect varying impacts on pathogens and LAB due to their different cell compositions. According to the product label, food preservatives such as E200, E211, E221, and E223 were added to some of them. Lactobacilli were detected to be more susceptible to phenolic acids than *E. coli* and *P. aeruginosa* [55]. The fact that phenols, as an antimicrobial, are naturally occurring compounds in grapes [56] may be the reason for the low amount of LAB in our hardaliye samples. Irwin et al. [57] reported that *Lactobacillus casei*, *L. rhamnosus*, and *L. plantarum* showed a higher susceptibility to sulfites and also that *S. thermophilus* was killed by sodium bisulfite after a few hours. *E. faecalis* is resistant to sodium nitrite, whereas *Bacteroides coprocola*, *Clostridium tyrobutyricum*, and *Bifidobacterium longum* are susceptible [58]. It was indicated that there were no food preservatives on the labels of samples C8, C17, C18, C22, C23, and C24. Thus, we may be able to relate this fact to more OTUs associated with the *Bacillus* genus detected in these products (Figure 5).

However, some unpasteurized samples (C4, C5, and C9) had higher diversity but less lactic acid bacteria (Figure 3 and Figure 5). The microbial diversity may have increased in these products since there was no pasteurization but it should be kept in mind that the absence of pasteurization may not always mean that it will increase the LAB number. There are multiple environmental factors, except heat processing techniques, that impact LAB viability [49]. The samples (C8, C17, C23, and C24) with the highest amount of *Bacillus* spp. are the ones that have not been treated with food preservatives or pasteurized (Figure 5). The results show that the lesser the food processing techniques used, or indeed the absence of food processing, the greater the viability of potential probiotics. Samples of C4 and C10 were identified as having the highest microbial content despite being processed with only food preservatives according to the label. Pasteurization may have a larger antimicrobial impact than food preservatives. However, having a diverse microbial composition does not necessarily imply that the product contains a high concentration of probiotics. For example, samples of C4, C5, C9, C10, and C20 showed an inverse trend between microbial diversity and LAB amount (Figure 2 and Figure 5).

The way that products are stored in markets, on shelves, and in cargo warehouses can change the viability of LAB and probiotics. If the food products are stored inappropriately on the shelves, the claimed probiotic populations may not be available to consumers [59]. As the fermented products remained on the shelves, amino acids, terpenoids, and polyketides were significantly boosted and bacterial growth may have declined. This fact suggests that the microbial community may be quite diverse between homemade and commercially packaged fermented foods due to the enzymatic activity of various microorganisms, environmental conditions, and human factors [20,21]. As our commercial product samples remain on the shelves longer than homemade products, it will be unsurprising to encounter low levels of LAB or probiotics. The packaged liquid samples reached the laboratory two to eight months after their production date.

Shipping temperatures are another issue to be discussed. The lower the temperature, the more stable the cells are [49]. The production processes of each of our samples are not clearly known. Online sales have increased during the pandemic period. It should not be forgotten that the storage and transfer conditions of the products received in this way may affect the product microbiome. For this reason, legal authorities related to the food safety chain in online sales, which continue rapidly, especially after the pandemic, need to switch to new inspection procedures in this regard.

Since some of our products claim to be probiotics, we would have expected to detect more probiotics or LAB. Ding and Shah [52] indicated that probiotic bacteria are expected to be at the level of 10^7^ CFU of live microorganisms per millilitre of product at the time of consumption. Even though the bacterial load was not tested in our investigation, we understood that some of our fermented products, such as hardaliye and kombucha, did not have diverse lactic acid bacteria. This is an interesting finding as it is contrary to what we see on the package of the brands. Fermented foods are associated with numerous health advantages and they are usually assumed to be full of probiotics. We believe that industrial-scale production requires the use of consistent microbes, fermentation processes, nutritional composition analysis, and food safety testing, as Baliyan et al. [60] declared.

Vegetables and fruits to be fermented can acquire pathogens in several ways, including through human intervention, post-harvest handling, cross-contamination by the washing of tanks and vehicles, and pre-harvest irrigation water composed of groundwater, surface water, and human wastewater. Phytopathogens and enteric pathogens may result in many biological risks, including crop losses and human diseases [61,62]. Previous studies showed the presence of many opportunistic pathogens in different food products. *Arsenophonus*, an emerging insect-vectored plant pathogen [63], was detected in hardaliye (2%) and kombucha (2%) samples; *Acinetobacter johnsonii*, bacteria that have a plant growth promotion effect [64], and *Shewanella* sp. Arc9-LZ, a metal-reducing bacteria [48], were found in hardaliye, kombucha, and grape vinegar samples.

Normally, there is an expectation of a negative correlation between the number of LAB and pathogens in fermented food. At the end of a proper efficient fermentation process, LAB levels are expected to increase so that they can inhibit the growth of food-borne pathogens [65]. In our study, the low number of potential LAB in hardaliye and kombucha products (Figure 5) may be the reason for more OTUs related to opportunistic pathogens. Our results led us to suspect the efficiency of fermentation in these commercial liquid products. The reason the probiotics were seen in lower amounts in hardaliye and kombucha could be the difference between the raw material and the fermentation technology, depending on the fermentation environment [21] (Figure 3). The study of Xie et al. [66] indicated that the most significant difference between foods collected at different stages is the quantitative ratios of each microbe, not the bacterial composition. Live bacteria, or probiotics, must be present in sufficient numbers for a product to be beneficial. Some commercial products can last several months at room temperature without losing more than one log of viability. Further specialized and controlled production conditions are needed in order to obtain such products [59].

**Figure 6:**
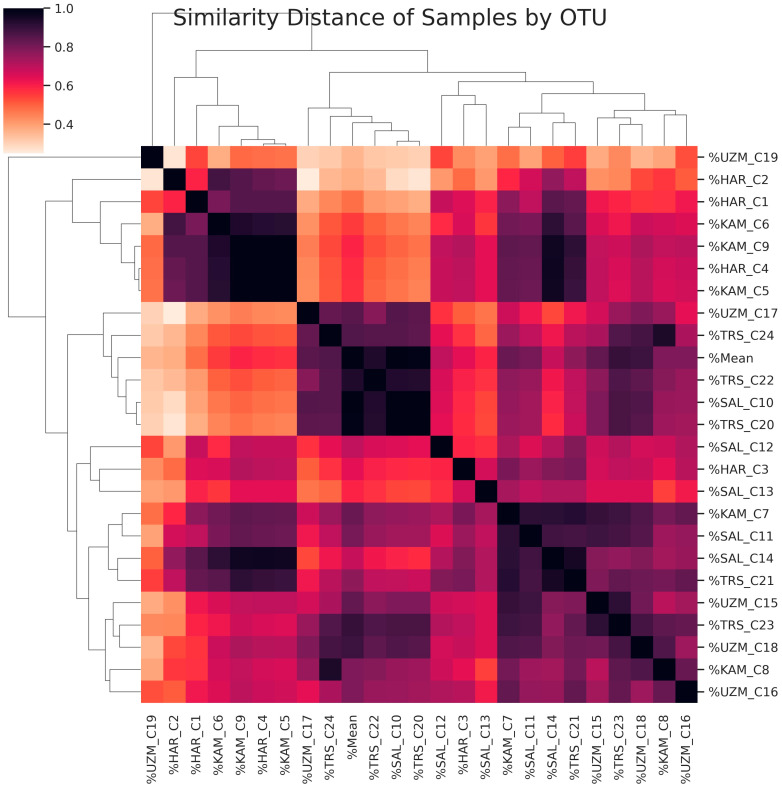
Genus similarity matrix. UZM: Grape Vinegar, HAR: Hardaliye, KAM: Kombucha, TRS: Pickle Juice, SAL: Shalgam Juice.

**Figure 7:**
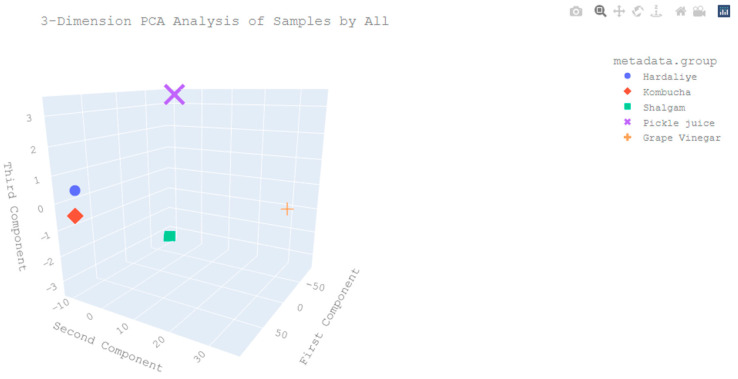
Three-dimensional principal component analysis (PCA) PCA (3D) ordination: PC1: First component along the x- and y-axes, PC2: Second component, PC3: Third component. Each group of products is represented by different coloured figures. The closeness of the samples to each other according to their microbial diversity is shown.

The closeness of the samples to each other biostatistically, according to their diversity, is shown in Figure 6 and Figure 7. Figure 6 shows the similarity relationship between genera based on Pearson similarity and the single linkage hierarchical clustering method. As the similarity ratio increases, the color becomes darker, so it approaches 1. According to Figure 7, the most striking result is that the pickle juice samples were clustered differently from the other groups.

## 4. Conclusions

The microbial diversity in some fermented liquid products, such as hardaliye, kombucha, shalgam juice, pickle juice, and grape vinegar, was screened comparatively, and consequently, it was determined that the probiotic diversity in some products was not as high as expected. Additionally, bacteria used in agriculture to support plant growth have also been encountered in our products. We think that these results will shed light on other studies and this may be important for the public’s health because live bacteria or probiotics must be present in sufficient numbers for a product to be beneficial. Therefore, some new production and storage techniques should be developed in order to encounter higher probiotic values in these products and to ensure the stability and viability of LAB and probiotics. Online sales have also increased during the pandemic period. With that in mind, we recommend increasing the control procedures or audits of commercial products since there are process differences between brands. Despite the fact that the items we deal with have manufacturing permits, the microbiological composition varies based on the brand due to prescription differences. Furthermore, this work demonstrated that pasteurization may have a larger antimicrobial impact than food preservatives and that having a diverse microbial composition does not necessarily imply that the product contains a high concentration of probiotics. It has been observed that the microbial content of each product is not the same. It is known that the roles of each bacterium in the body are different. Therefore, we suggest that it is beneficial to include different fermented liquid products in our diet.

## Figures and Tables

**Table 1:** Sampling Plan and Size.

Fermented Products	Sample Code	Number of Subsamples	Production Process Applicatıon Type *		Number of Total Subsamples
			Food Additive	Pasteurization	
Hardaliye	C1	4	YES	NO	16
	C2	4	YES	YES	
	C3	4	YES	YES	
	C4	4	YES	NO	
Kombucha	C5	4	NO	NO	20
	C6	4	NO	NO	
	C7	4	NO	NO	
	C8	4	NO	NO	
	C9	4	NO	NO	
Shalgam Juice	C10	4	YES	NO	20
	C11	4	NO	NO	
	C12	4	NO	YES	
	C13	4	YES	YES	
	C14	4	YES	NO	
Grape Vinegar	C15	4	YES	NO	20
	C16	4	NO	NO	
	C17	4	NO	NO	
	C18	4	NO	YES	
	C19	4	YES	YES	
Pickle Juice	C20	4	NO	NO	20
	C21	4	NO	YES	
	C22	4	NO	NO	
	C23	4	NO	NO	
	C24	4	NO	NO	
				Number of Total Samples	96

* Indicates the type of food process application as stated on product labels.

**Table 2:** The relative abundance of plant growth regulator or promoting bacteria in our samples.

Plant Disease Prevention and Plant Growth Regulation Bacteria According to the Literature	The Relative Abundance of Plant Growth Regulator or Promoting Bacteria in Our Samples				
	Hardaliye	Kombucha	Shalgam Juice	Pickle Juice	Grape Vinegar
*Actinobacteria* [38]	3	2.4	1.5	1.5	-
*Arthrobacter* [27]	1	-	-	-	-
*Klebsiella* [39]	1.4	1.5	-	-	-
*Paenibacillus mucilaginosus* [36]	-	-	15.5	14.2	3
*Paenibacillus barcinonensis* [36]	-	-	8.9	8.8	3
*Enterococcus faecium* [40]	-	-	9.7	12	3
*Bacillus* spp. [27]	1.5	1.5	14	14.1	14.7
*Bacillus paralicheniformis* [41]	-	-	-	-	11.76
*Bacillus velezensis* [42]	-	-	3	3	-
*Collimonas fungivorans* [43]	6.1	6.1	1	-	2.9
*Pseudomonas stutzeri* [44]	5	5	1.1	-	-
*Acinetobacter* spp. [45]	4.4	4.3	-	-	2.9
*Burkholderia thailandensis* [46]	1.9	1.6	-	-	-
*Salmonella enterica* [47]	3.8	4.2	-	-	-
*Shewanella* sp. Arc9-LZ [48]	1.1	1.3	-	-	3

-: Results were detected with less than 1% or not detected.

## Data Availability

The data used to support the findings of this study can be made available by the corresponding author upon request.
